# Short-Term PTEN Inhibition Improves *In Vitro* Activation of Primordial Follicles, Preserves Follicular Viability, and Restores AMH Levels in Cryopreserved Ovarian Tissue From Cancer Patients

**DOI:** 10.1371/journal.pone.0127786

**Published:** 2015-05-29

**Authors:** Edurne Novella-Maestre, Sonia Herraiz, Beatriz Rodríguez-Iglesias, César Díaz-García, Antonio Pellicer

**Affiliations:** 1 Unidad de Genética, Hospital Universitario y Politécnico La Fe, Valencia, Spain; 2 Grupo de investigación de Medicina Reproductiva. Instituto de Investigación Sanitario La Fe. Valencia, Spain; 3 Unidad de Preservación de la Fertilidad, Área de Salud de la Mujer. Hospital Universitario y Politécnico La Fe, Valencia, Spain; 4 IGENOMIX, Parc Cientific Valencia University, Paterna, Valencia, Spain; Qingdao Agricultural University, CHINA

## Abstract

**Introduction:**

*In vitro* activation and growth of primordial dormant follicles to produce fertilizable oocytes would provide a useful instrument for fertility preservation. The employment of Phosphatase and TENsin homolog (PTEN) inhibitors, in combination with Protein kinase B (Akt) stimulating molecules, has been previously employed to increase follicular activation through the stimulation of the PTEN-Akt pathway.

**Methods:**

We aim to establish improved *in vitro* activation also for cancer patients whose ovarian tissue has already been cryopreserved. Fresh and previously cryopreserved human ovarian cortex were exposed to short-term, low-concentration and ovary-specific treatment with only a PTEN inhibitor.

**Results:**

Our *in vitro* activation protocol enhances the activation mechanisms of primordial follicles in both fresh and cryopreserved samples, and enlarges growing populations without inducing apoptosis in either follicles or the surrounding stroma. Treatment augments estradiol secretion and restores the expression levels of the previously diminished Anti-Müllerian hormone by means of cryopreservation procedures. Genomic modulation of the relative expression of *PTEN* pathway genes was found in treated samples.

**Conclusion:**

The *in vitro* activation protocol offers new alternatives for patients with cryopreserved tissue as it increases the pool of viable activated follicles available for *in vitro* growth procedures. The combination of ovarian tissue cryopreservation and *in vitro* activation of primordial follicles, the main ovarian reserve component, will be a major advancement in fertility preservation.

## Introduction

Survival after cancer in adolescence and childhood has improved [1]. Thus a large population of young women, who have not fulfilled their reproductive project, will suffer secondary effects of cancer treatment, such as gonadotoxicity. This circumstance, together with the fact that our society delays childbearing age [2], means that fertility preservation (FP) is being increasingly requested, especially in young cancer patients.

Several options are currently available for female FP, such as cryopreservation of oocytes [3], embryos [4], or ovarian tissue [5–7].

The ovarian cortex contains quiescent primordial follicles characterized by their resistance to freezing and thawing processes. Given these properties, the cryopreservation of ovarian cortex for subsequent autologous orthotransplantation is the most widely used technique to preserve fertility in cancer patients [8]. Furthermore, it is the only option in pediatric patients with no mature oocytes to be cryopreserved, and for cases of hormone-dependent diseases [9, 10].

The total number of available primordial follicles is, among other important questions, the main determinant to ensure FP success. That can be compromised by several factors, such as ovarian cortex size, age, previous chemotherapy, and other potential effects of cancer on female gonads. It has been already published that malignancies, such as breast cancer, can also affect reproductive outcome as ovarian reserve is impaired in young women with germline *BCRA1* mutations [11]. Ovarian response to controlled stimulating cycles diminishes in cancer patients, even before they receive any treatment [12]. Nevertheless, the risk of reintroducing malignant cells into transplanted tissue is the main concern of ovarian cryopreservation. This adverse event is at increased risk [13–15] in patients with hematologic cancers, such as leukemia, the commonest malignancy of childhood [16]. Therefore, new safe alternatives should be developed to optimize ovarian reserve in these patients where cryopreservation and transplant of ovarian cortex are contraindicated [17], or in pediatric patients who have no mature oocytes to be cryopreserved [18].

As a previous step to growth, primordial dormant follicles, the main ovarian reserve component [19, 20], have to be activated for their developmental program to start. Diverse pathways are involved in follicle activation guidance through the control of oocyte growth initiation and maintenance, such as the Phosphatase and tensin homolog deleted on chromosome 10 (PTEN), phosphatidylinositol 3 kinase (PI3K), forkhead box O3 (FOXO3), and the mammalian target of rapamycin complex 1 (mTORC1) [21–26]. Nevertheless, the underlying mechanisms of activation remain unknown.

It has been reported that the growth of all primordial follicles in neonatal and adult animals is promoted by the oocyte-specific deletion of the *PTEN* gene [21, 24–26]. This gene encodes a phosphatase enzyme that negatively regulates the PI3K-Protein kinase B (Akt) signaling pathway. *PTEN* deletion increases Akt phosphorylation and the nuclear export of downstream FOXO3 proteins [24]. Indeed *FOXO3* gene deletion also activates all dormant follicles in mice. Recently, Li et al. 2010 [26] developed a short-term, ovary-specific treatment of rodent and human ovaries with a PTEN inhibitor and/or a PI3K activator. Treatment increased FOXO3 nuclear extrusion in primordial oocytes, which led to their activation.

Based on these findings, a clinical trial was carried out by Kawamura [27] to investigate the effectiveness of the PTEN inhibitors and Akt stimulator molecules used for the *in vitro* activation (IVA) procedure in women with premature ovarian failure. This study proved that the remaining quiescent follicles of ovarian reserve can be rescued by inducing activation mechanisms to produce fertilizable oocytes.

Encouraging by these results, the aim of this study was to achieve the activation of human dormant primordial follicles immersed in the ovarian cortex from cancer patients through incubation with a PTEN inhibitor to avoid oocyte degradation to, therefore, increase the "pool" of viable primordial follicles available for *in vitro* growth procedures.

## Material and Methods

### Chemicals

All the chemicals, culture media and reagents used in this study were purchased from Sigma-Aldrich (St. Louis, MO, USA), unless otherwise stated.

### Study design

#### Ethics statement and tissue collection

This study was approved by the Institutional Review Board of Hospital Universitario y Politécnico La Fe, Valencia, Spain (2011/0018) and conducted according to the principles expressed in the Declaration of Helsinki. After obtaining written informed consent, 18 human ovarian cortex biopsies from the women recruited in our Fertility Preservation Program were obtained. Ovarian cortex biopsies were only included if the remaining tissue was available after the ovarian cortex collection for fertility preservation purposes. Patients were affected by breast cancer (n = 8), rhabdomyoma (n = 1), acute lymphoblastic leukemia (n = 1), Hodgkin lymphoma (n = 4), anti-synthetase syndrome (n = 1), Ewing´s sarcoma (n = 1), medulloblastoma (n = 1) and non Hodgkin lymphoma (n = 1). The mean patient age was 27.8 (range 14–37) years. No patient had undergone chemo/radiotherapy before ovarian cortex extraction. Ovarian biopsies (an approximate thickness of 10x10x1 mm) were immediately washed with serum-free M199 medium at 37°C, dissected by removing medulla and cut into two pieces: one to test the IVA protocol in fresh tissue and the other to test it in cryopreserved/thawed tissue. Due to the limited sample size, only 14 of the 18 obtained biopsies were included in the cryopreserved group.

#### Experimental groups

Fresh ovarian pieces (n = 18) were divided into strips; one was immediately fixed to act as an initial condition fresh control tissue and was allocated to Group 1 (S1 Fig); the other was allocated to Group 2 and was *in vitro* activated and cultured for 25 h with the PTEN inhibitor.

In order to assess whether there were any deleterious effects due to culture conditions, additional fresh ovarian strips (n = 9) were allocated to Group 3 and were incubated under the same conditions as the activated ones, but without the PTEN inhibitor to act as a fresh cultured control.

Cryopreserved/thawed ovarian pieces (n = 14) were also divided into strips; one was fixed immediately after thawing to act as an initial condition cryopreserved control tissue and was named as Group 4; another was allocated to Group 5 to be incubated and cultured with the PTEN inhibitor for 25 h; a third one was cultured for 25 h without the PTEN inhibitor and was considered the cryopreserved cultured control, called Group 6.

The samples from the fresh and cryopreserved cultured control groups (Groups 3 and 6) were used for the TUNEL assay and the hormone production quantification by ELISA, as described below.

#### Ovarian Tissue Cryopreservation and Thawing

A slow-freezing technique was used to cryopreserve ovarian strips. M199 culture medium, containing 5% Human Serum Albumin (HSA) and 10% of Dimethyl sulfoxide (DMSO), was employed as a cryoprotectant solution. DMSO was sequentially added in a 2-step procedure and fragments were distributed in ethyl vinyl acetate bags (Cryocyte, Baxter Healthcare, Deerfield, IL, USA). Sealed bags were placed in the freezing chamber of a controlled-rate freezing device (Planer). A slow-freezing protocol was applied following the same procedure currently employed for clinical purposes in our fertility preservation program. Bags were stored in liquid nitrogen (-196°C), as previously described [28, 29]. Thawing took place after 24 h. Bags were thawed and cryoprotectant was eluted using a 3-step protocol, as described elsewhere [28, 29].

### IVA protocol

Short-term incubations with Dipotassium Bisperoxo (picolinato) oxovanadate V, PTP Inhibitor XV (bpV(pic)), were performed as an IVA protocol. Briefly, activated samples were first incubated for 1 h with 100 μM of bpV(pic) (Calbiochem, Merck KGaA, Germany) in α-MEM medium supplemented with 10% HSA and 1% Antibiotic-Antimycotic Solution (ATB). Then samples were incubated for 24 h with the same media (100 μM bpV(pic)+ 10%HSA, 1% ATB- α-MEM) supplemented with 0.3 I.U of FSH. All the incubations were performed under standard culture conditions at 37°C, 5%CO_2._

In the cultured control groups (G3 and G6), samples were incubated under the same conditions, but bpV(pic) was not added to the culture medium. After the IVA procedure and culturing, samples were fixed in neutral buffered formalin and the culture medium was immediately stored at -80°C for hormone quantification.

### Histological evaluation and follicular counts

Formalin-fixed samples were paraffin-embedded (FFPE) and cut into 4-μm thick sections. Every 10^th^ section was stained with hematoxylin-eosin (H&E) to perform the histological analysis and follicular counts. The remaining sections were used for the immunohistological analysis.

In the H&E-stained samples, follicles were classified as follows: Primordial stage: the oocyte was surrounded by a layer of flattened granulosa cells (GC); Primary stage: the oocyte was surrounded by a complete layer of cuboidal GC; Secondary stage: the oocyte was surrounded by two layers, or more, of cuboidal GC [19]. Follicles were counted only when the oocyte nucleus was present to avoid double counting. Only healthy follicles [30] were counted to establish the densities and percentages of the quiescent (primordial) and growing follicles (primary, secondary). All the H&E-stained sections were examined by two different observers (ENM, SH).

### Immunohistochemistry

To establish follicular activation, proliferative status and growth through secondary stages, immunostaining with FoxO3a, Ki-67 and Anti-Müllerian hormone (AMH) was done. A rabbit monoclonal anti-human FoxO3a antibody (Cell Signaling Technology, Inc. Danvers, MA, USA), at the 1:150 dilution, was incubated for 60 min at room temperature (RT) to detect follicle activation. A monoclonal mouse anti-human Ki-67 antibody (MIB-1, Dako Denmark A/S, Glostrup, Denmark), at the 1:100 dilution, was incubated at RT for 60 min, and was employed to specifically detect follicular proliferating cells. A goat polyclonal anti-human-MIS antibody (Santa Cruz Biotechnology, Inc. Heidelberg, Germany), at the 1:200 dilution, was used to detect AMH (60 min and RT). For all the antibodies, a biotin/streptavidin reaction was used for the secondary antibody incubation (LSAB method Dako Denmark A/S, Glostrup, Denmark), followed by detection with 3,30-diaminobenzidine (DAB). For the negative controls, the primary antibody was omitted; for the positive controls an additional slide, containing lung, proliferative endometrium and testis, was included.

Follicles were considered activated when the FoxO3 nuclear extrusion on the oocyte was detected. Ki-67 and AMH were positive when detected in GC and in oocyte nuclei/GC, respectively.

### AMH and Estradiol (E2) production

For AMH determination, the culture media samples were analyzed by AMH-Gen-II ELISA (Beckman Coulter, Immunotech, Texas, USA) following the manufacturer´s protocol. Briefly, samples and controls were dispensed into the micro-titrated wells coated with the anti-AMH antibody. After incubation and washing, the biotin-labeled anti-AMH detection antibody was added. After washing, streptavidin-horseradish peroxidase was added to the wells and developed. Then absorbance was measured in an automatic ELISA reader (Bio-Rad, Hercules, CA, USA). The limit of detection (LOD) of the AMH assay was 0.08 ng/ml; this assay does not detect inhibin A, activin A, FSH and LH. The intra- and inter-assay variations were 3.7% and 4.4%, respectively.

E2 was measured with the Estradiol EIA kit (Cayman Chemical Company, Michigan, USA). The assay was performed following the manufacturer´s protocol in undiluted samples. Absorbance was measured at 405–420 nm. The LOD of the E2 assay was 20 pg/ml, and interference with other steroid hormones was < 0.01%. For 102.4 pg/ml, the intra- and inter-assay variations were 13.0% and 8.2%, respectively.

In both assays, absorbance was inversely proportional to the concentrations of AMH and E2 in the samples, which was calculated from the calibration curve. Samples were run in triplicate. The results were expressed in ng/ml and pg/ml, respectively, and also according to the established standard curve.

### Apoptosis quantification by TUNEL

DNA fragmentation was detected by the TdT (terminal deoxyribonucleotidyl transferase)-mediated dUTP nick-end labeling (TUNEL) assay using the TMR red *in situ* cell death detection kit (Roche Diagnostics, Germany). Three paraffin-embedded sections per sample were cleared with xylene and rehydrated (ethanol, distilled water) prior to being assayed. After washing with PBS, antigen retrieval with 10 mM citrate buffer was performed in a microwave for 5 min. Then slides were incubated for 60 min at 37°C inside a dark humidified chamber with 50 μl of the TUNEL reaction mixture; this mixture was omitted in the negative control. Samples were mounted with ProLong Gold Antifade Reagent with DAPI (Life Technologies Inc., Gaithersburg, MD, USA) and examined under a microscope by conventional fluorescence. The apoptotic signal was recorded as positive when dUTP stained the nucleus red.

Cell damage was quantified in primordial follicles, the main ovarian reserve component, and in the ovarian stroma. Follicles were considered damaged when the oocyte nucleus, or more than 50% of GC, was stained red by dUTP. In the ovarian stroma, the cell death investigated by TUNEL was expressed as Percentage of Cell death [(TUNEL+ cells/total cells)*100]. The apoptotic rate of the IVA groups (G2 and G5) was compared with their respective cultured controls (G3 and G6). The selection of the cultured controls allowed us to elucidate if the cell damage, when observed, could be due to the PTEN inhibition or just to the culture conditions. Furthermore, the apoptotic grade detected in fresh ovarian cortex or induced by cryopreservation techniques, has been previously established [28, 31–34].

To quantify the TUNEL assay, high-resolution images were obtained by light microscopy (LEICA DM4000B, Leica Microsystems GmbH, Germany) with a digital camera attached (LEICADFC450C, Leica Microsystems GmbH, Germany). Quantification was assessed by the Image ProPlus 6.3 software (Media Cybernetics Inc. Rockville, MD, USA).

### Relative gene expression of the PTEN pathway

mRNA was obtained from 7–9 FFPE ovarian samples of each group with the Recover All Total Nucleic Acid Isolation Kit (Ambion, Life Technologies Inc., Gaithersburg, MD, USA) following the manufacturer´s protocol. For each sample, four 15-μm slides were used. Then cDNA was synthesized with MultiScribe Reverse Transcriptase from the High-Capacity cDNA Reverse Transcription Kit (Applied Byosistems, Life Technologies Inc., Gaithersburg, MD, USA). Specific Taqman probes were assayed by Real-Time PCR to quantify the relative expression of the following genes: *PTEN*, *PI3KCB*, *AKT1* and *FOXO3*. PCR reaction mixtures were prepared with 100 ng of cDNA as a template in the 1xTaqman Gene Expression Master Mix following the manufacturer´s protocol. Then samples were amplified in the 7900HT Fast PCR system (Applied Byosistems, Life Technologies Inc., Gaithersburg, MD, USA). PCR conditions consisted in an initial activation of uracyl-*N*-glycosylase at 50°C for 2 min., followed by AmpliTaq Gold activation at 95°C for 10 min. Then samples were cycled 40 times by denaturation at 95°C for 15 seconds, and an annealing extension at 60°C for 1 min per cycle. Each sample was assayed in triplicate.

After controlling the amplification efficiency of the targets genes, the relative expression was obtained by the comparative Ct method (DDCt) [35]. The expression of target gene mRNA was normalized with the expression of the 18S reporter gene.

### Statistical analysis

All the data are presented as mean ± SEM. The Friedman test, followed by a Wilconox paired *post hoc* test, was performed to evaluate the results between groups. p<0.05 values were considered statistically significant. All the analyses were performed with SPSS 19.0 (IBM, Somers, NY, USA).

## Results

### Follicular densities and populations

Among the H&E-stained ovarian sections, 597 follicles were counted and examined. When primordial, primary and secondary densities were compared among the activated samples and their respective initial condition controls (G1 and G4), no significant differences were detected in the fresh or cryopreserved samples (p = ns), nor when they were compared with the cultured control groups (G3 and G6) (Table 1).

**Table 1:** Follicular densities and percentages.

	Ovarian tissue treatments			Follicular density (foll./mm^2^)			Follicular populations (%)	
	Cryop./ Thaw	IVA	Culture period	Primordial	Primary	Secondary	Quiescent	Growing
**G1**	N	N	0h	1.18±0.46	0.18±0.06	0.01±0.01	62.3±8.3	37.7±8.3
**G2**	N	Y	25h	2.5±1.7	0.5±0.2	0.06±0.03	47.9±8.2	52.0±8.2
**G3**	N	N	25h	0.78±0.21	0.40±0.21	0.04±0.03	61.7±12.2	38.2±12.2
**G4**	Y	N	0h	1.7±1.2	0.4±0.1	0.02±0.01	55.0± 6.1	46.9 ±6.1
**G5**	Y	Y	25h	0.7±0.3	0.4±0.09	2.6±2.6	38.2±7.1^a^	61.9±7.5^b^
**G6**	Y	N	25h	0.59±0.19	0.27±0.07	0.01±0.01	49.69±8.9	50.3±8.9

^a^ G4 *vs*. G5 quiescent follicle population, p = 0.02.

^b^ G4 *vs*. G5 growing follicular population, p = 0.03.

The experimental interventions performed on the ovarian tissue samples included in each experimental group of our study are listed in Table 1. Follicular densities and the percentages of each population were calculated. The values are expressed as mean ± SEM. The cryopreserved activated (G5) group showed statistically significant differences for the percentage of quiescent and growing follicles compared to the initial condition.

However after activation, the percentages of primordial follicles lowered in both the fresh and cryopreserved IVA groups when compared with the initial condition controls of tissue (Group 1: 62.3±8.3%; G2: 47.9±8.2%; G4: 55.0±6.1%; G5: 38.2±7.1%), but the percentages of the growing ones increased (G1: 37.7±8.3%; G2: 52.03±8.2%; G4: 46.9±6.1%; G5: 61.9±7.1%) when compared to their controls, or when compared with the cultured controls (G3 and G6). This phenomenon was statistically significant only in the G5 group (p = 0.02 and p = 0.03, respectively).

In order to elucidate if there was any difference between our immediately fixed control and the cultured controls, an exploratory study was performed (S1 Table). No differences were detected between the fresh and cryopreserved initial condition control tissues when primordial, primary and secondary follicular densities were compared with their respective cultured control tissues, nor when the quiescent and growing populations were compared.

### Follicular activation

**Fig 1:**
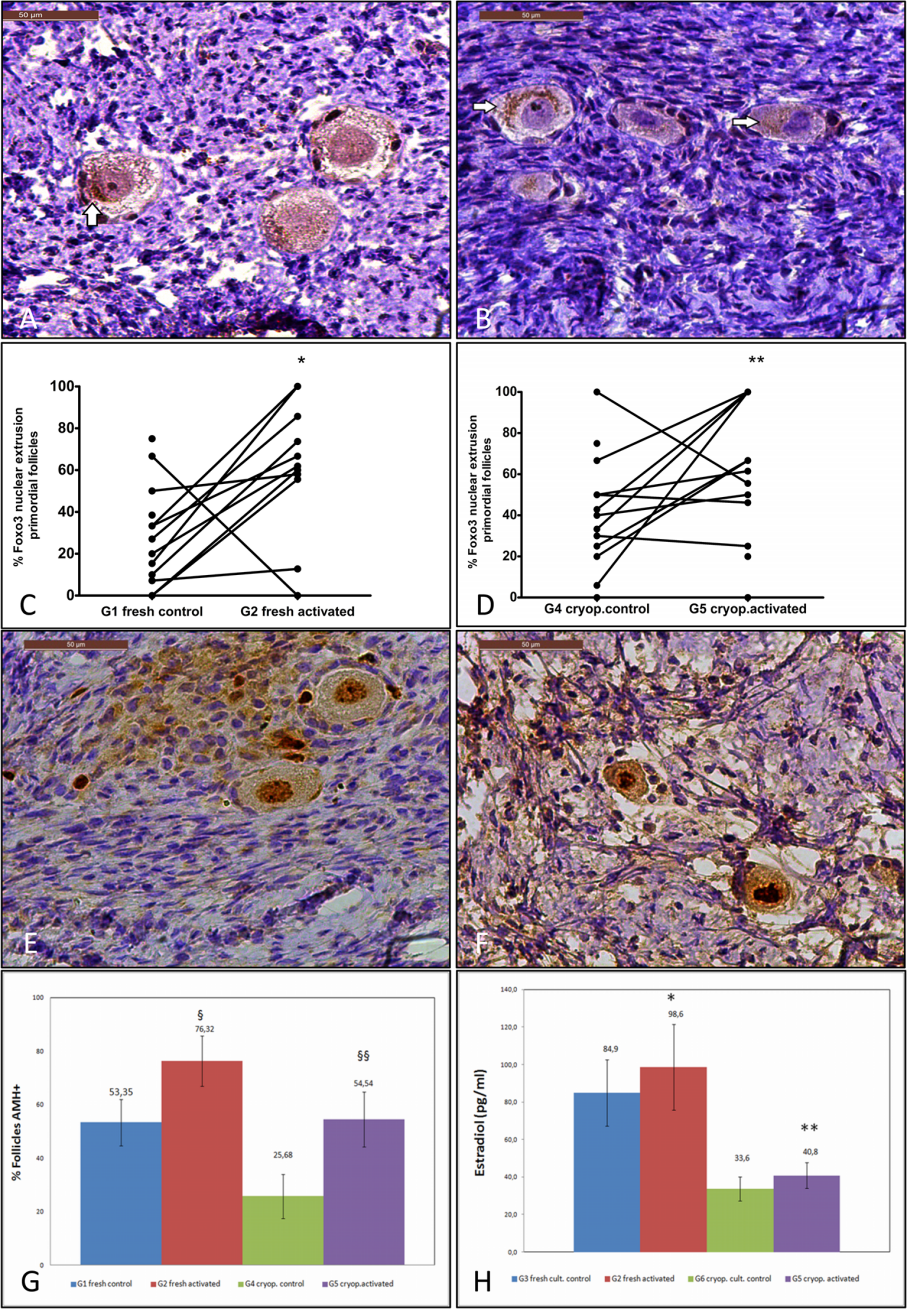
Activation, proliferation and hormone production induced by IVA. (A) and (B); FOXO3 detection and localization to monitor follicular activation. Follicles from the G2 and G5 samples are shown, respectively. Note that the activated follicles from the G2 and G5 groups present a FOXO3 nuclear extrusion (arrow) and a positive signal in GC; (C) the percentage of primordial follicular activation was assessed in fresh ovarian tissues, and a significant increase was observed in G2 when compared to G1 (*p = 0.03); (D) when the activated primordial follicular percentage was compared in the previously cryopreserved-thawed group, follicle activation was enhanced in G5 vs. G4 (**p = 0.03); (E) fresh activated sample (G2), primordial follicle showing Ki-67 staining in GC and oocyte nuclei; F) the Ki-67-positive signal in the oocytes of the primordial follicles from the cryopreserved-activated sample (G5); (G) quantification of the AMH expression in GC. An increase in the AMH expression was observed in the GC from the follicles in both G2 (§ p = 0.006) and G5 (§§ p = 0.008) as a result of the *in vitro* activation procedure with 100 μM of bpv(pic); (H) estradiol secretion to culture media. As the graph depicts, *in vitro* activation procedures increased E2 secretion in the *in vitro* activated fresh (G2 *vs*. G3 *p = 0.036) and cryopreserved (G5 *vs*. G6 **p = 0.001) samples when compared to their own controls.

FOXO3 nuclear export, an indicator of follicle activation, was monitored (Fig 1A and 1B) and quantified (Fig 1C and 1D) in the activated samples, and also in their respective controls. A 3-fold increase in the percentage of activated primordial follicles was seen in the fresh activated samples (G2: 59.55±9.88%) when compared to their initial control (G1: 23.69±5.71%; p = 0.03). When primary follicle activation was analyzed, it was not statistically significant despite the 15% increase observed in the activated group (G2: 80.44±4.8%, G1: 66.4±8.9%, p = NS). In the cryopreserved activated samples (G5), the IVA protocol produced a significant 2-fold increase in primordial and primary follicle activation (Fig 1D) when compared with their control group (Primordial: G5: 63.7± 8.9%; G4: 36.8±7.2%, p = 0.04 and primary: G5: 93.80±2.4%; G4: 47.5±8.9%; p = 0.03).

### Proliferation and growth to secondary stages

The Ki-67-positive cells were detected in oocytes and GC from all the activated samples (Fig 1E and 1F). This finding revealed that bpv(pic) exposure, even after the cryopreservation procedures, does not affect the proliferative capability of primordial and primary follicles.

AMH immunostaining revealed a significant increase, of around 23–30%, in the AMH positive follicles after IVA treatment in the activated samples from groups G2 and G5 when compared to the initial condition controls of the tissue (G1: 53.3±8.7% *vs*.G2: 76.3±9.4%; p = 0.006 and G4: 25.7±8.2% *vs*. G5: 54.5±10.2%; p = 0.008) (Fig 1G).

### AMH and Estradiol production

Quantification of AMH in culture medium showed that the IVA protocol significantly increased the AMH concentration in the fresh activated samples when compared to the cultured control group (G2: 0.34±0.1 ng/ml *vs*. G3: 0.47±0.1 ng/ml; p = 0.017). However, this increase was not detected in the previously cryopreserved samples, where the AMH levels remained below the low LOD in all the analyzed samples.

A significant increase in the E2 concentration secreted to the culture medium was detected in both the fresh (G3: 84.9±17.7 pg/ml *vs*. G2: 98.6±22.9 pg/ml; p = 0.036) and cryopreserved IVA groups (G6: 33.6±6.3 pg/ml *vs*. G5: 40.8±6.8 pg/ml; p = 0.001) when compared to their respective cultured controls (Fig 1H).

### Apoptosis by TUNEL

**Fig 2:**
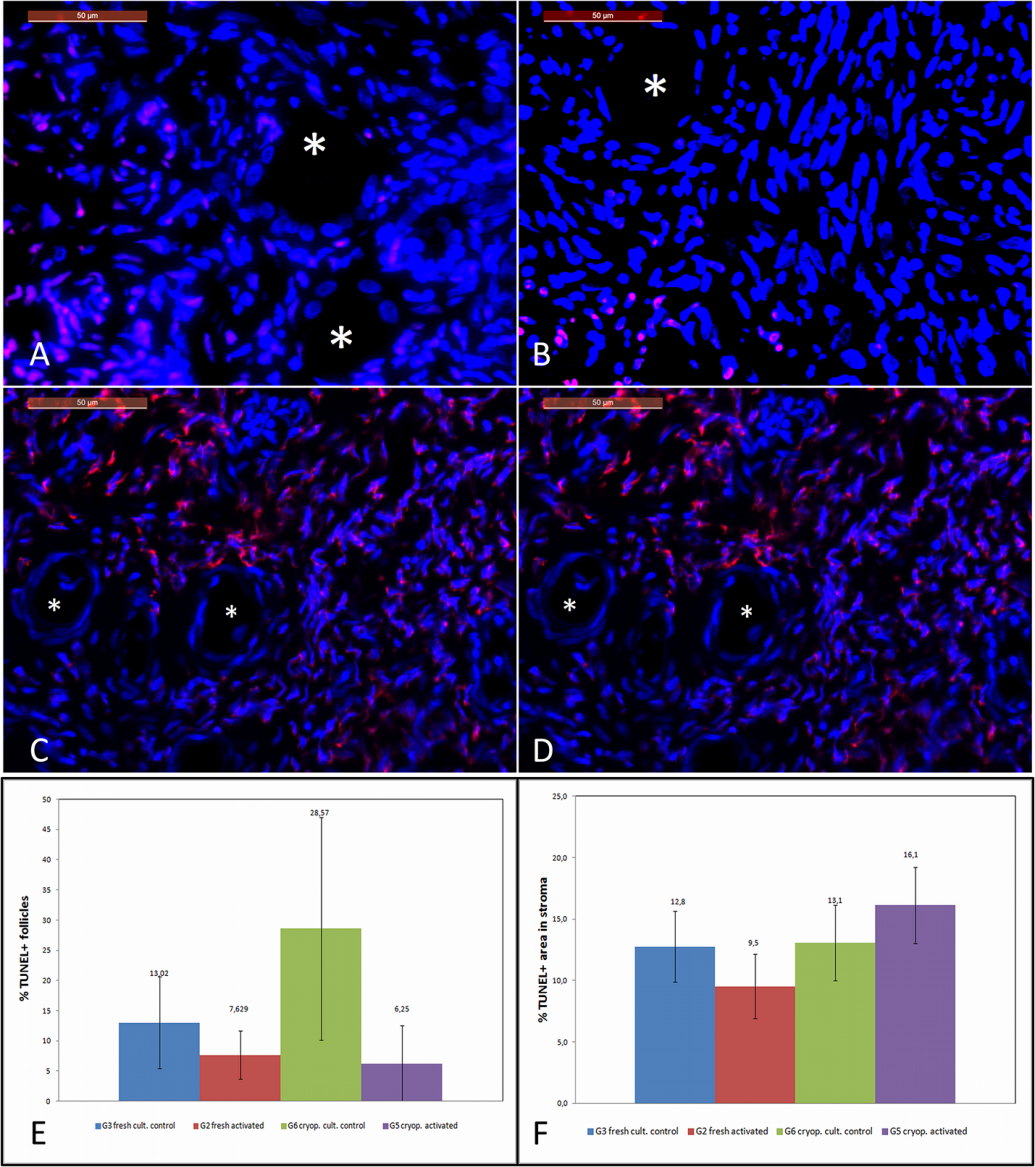
Apoptosis quantification by TUNEL analysis. (A) cell nuclei stained with DAPI (blue) to perform the TUNEL assay. The sample from the G3 group. The TUNEL + signal was found in the stoma cells (red) from G3; (B) note the primordial follicle in the G2 sample. The TUNEL + signal was found in the stroma, but note that it was absent in the oocyte and GC from the follicles of the G2 group; (C) the G6 sample. Primordial follicles surrounded by flattened shaped GC and stroma nuclei stained with DAPI (blue). TUNEL-positive cells stained in red in the stroma. Follicles were negative for DNA damage in the G6 sample; (D) the G5 sample that contained two primordial follicles, cell nuclei stained with DAPI. No TUNEL + signal was detected in oocyte and GC, but was positive in the ovarian stroma; (E) cell death index. Quantification of the follicles with a positive TUNEL signal in oocytes or GC. No differences were detected between activated and non activated samples; (F) TUNEL-positive stromal area quantification. No differences were detected for follicles when the TUNEL+ area was compared between the activated and non activated groups. This finding suggests that the cell damage found in the stroma from all the groups was due to the culture process.

To elucidate whether the IVA protocol induced any deleterious effect on the ovarian stroma or follicles, cell damage was investigated and quantified by TUNEL in the activated and control-cultured samples. TUNEL-positive cells were detected in all the experimental groups, even in both the cultured control groups, due to the culture conditions (Fig 2A to 2D). Regarding follicular cell damage (Fig 2E), the study showed that there was no statistical difference in the degree of damaged follicles between the activated groups and their respective culture control samples in the fresh (G2: 7.7±4.0% *vs*. G3: 13.0±7.6%; p = NS) and cryopreserved tissues (G5: 6.25±6.0% *vs*. G6: 28±18.4%; p = NS), although a broad variability between samples was detected (S2 Fig). When cell damage was quantified in the stroma, no significant differences due to the activation procedures were found (Fig 2F).

### Relative gene expression of the *PTEN* pathway

To validate the effect of bpV(pic) incubations on the gene expression of the *PTEN* pathway, the relative expression of the main genes was quantified.

**Fig 3:**
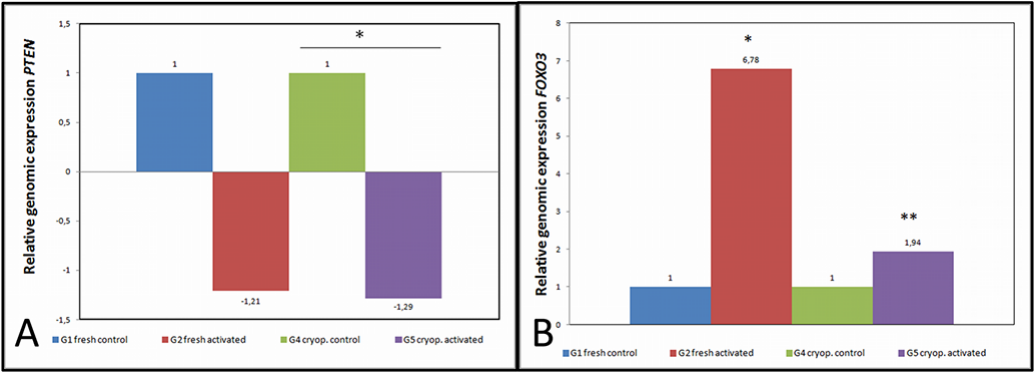
Analysis of the relative expression of the PTEN pathway genes. (A) fold change of the *PTEN* gene. Significant differences were obtained when ∆Ct were analyzed and showed the PTEN inhibition produced by bpv(pic) at 100 μM in fresh (G1 *vs*. G2 p = 0.01) and in previously cryopreserved ovarian tissues (G4 *vs*. G5 p = 0.04); (B) fold change of the *FOXO3* gene. A significant decrease was detected only in the fresh tissues that underwent IVA treatment when ∆Ct were analyzed (G1 *vs*. G2 p = 0.015).

In fresh tissues, the IVA protocol induced several significant modifications, such as a reduced fold change (fc) of the *PTEN* (fc = -1.21, p = 0.03) and *PI3K* (fc = -2.43) genes, and an overexpression of *AKT* (fc = 7.65, p = 0.015) and *FOXO3* (fc = 6.78, p = 0.01) in comparison to the controls (Fig 3A and 3B). When the Delta Cycle Threshold (ΔCt) was statistically compared between the activated and control samples (Table 2), significant differences were also noted among the *PTEN*, *PI3K* and *FOXO3* gene expression profiles (p = 0.01; p = 0.03 and p = 0.015, respectively).

**Table 2:** Gene expression analysis.

	∆Ct			
Group	*PTEN*	*Akt*	*PI3K*	*FOXO3*
G1	9.06±1.5	7.88±1.27	11.39±1.76	6.51±1.33
G2	11.73±0.56*	9.40±0.56	14.26±1.01*	9.25±0.79*
G4	10.49±1.45	8.41±1.31	11.72±1.63	8.08±1.53
G5	12.56±0.38*	9.82±0.48	13.53±0.54	9.35±0.56

∆Ct values were calculated and expressed as mean ± SEM.

(*) marks statistically significant differences in gene expression when compared to their respective control group.

For the cryopreserved samples, the relative expression or fold change of the *PTEN* pathway genes was also modified by IVA *(PTEN* fc = -1.29, *PI3K* fc = 0.59, *Akt* fc = 2.27,p = 0.007; and *FOXO3* fc = 1.94, p = 0.008) when compared to the controls (Fig 3A and 3B). Although the same expression pattern was observed, statistically significant differences were detected only for the *PTEN* gene when comparing ΔCt (p = 0.04).

Additionally, an exploratory study for gene expression was performed between initial condition samples and their respective cultured control. As S2 Table shown, no statistically significant differences were detected between control groups when compared with a paired test.

## Discussion

The use of an IVA protocol with a PTEN inhibitor in human cryopreserved ovarian cortex from cancer patients increased the pool of viable activated primordial follicles without inducing deleterious effects, as the obtained results indicate. This approach represents a major improvement in the first steps of *in vitro* growth techniques. It attaches particular importance to: those cancer patients whose ovarian cortex is cryopreserved as the only option for FP, but for whom direct tissue reintroduction is contraindicated [9, 10, 18]; those women whose ovarian reserve available to undergo *in vitro* growth and maturation procedures is reduced due to the limited amount of cryopreserved tissue; those in whom malignancy [11, 12], or even the cryopreservation procedure, depleted the follicular pool [28].

The safety of PTEN and Akt modulators has been well demonstrated in both rodent and human tissues. First in mice, in which healthy offspring have been obtained after activating the primordial follicles from neonatal mouse ovaries by transient treatment with PTEN inhibitor bpV(HOpic) [36]. Second in humans, in whom the IVA protocol proposed by Kawamura [27], which uses a combined treatment with a PTEN inhibitor and Akt stimulator molecules followed by *in vivo* oocyte development, resulted in the birth of a healthy boy. In contrast, a recent study published by McLaughlin [37] showed that although follicular exposure to 1 μM of bpv(HOpic), a PTEN inhibitor, increased the *in vitro* activation rate of primordial follicles, compromises growth and survival in these follicles when assessed the secondary stage. These discrepancies could be due to several factors, such as the PTEN inhibitor employed or the tissue preparation procedure applied prior to activation. Although bpv(HOpic) was used at a lower concentration, it presented high affinity and a low half maximal inhibitory concentration (IC_50_) than bpv(pic) [38]. This fact can affect follicular outcome after IVA as the *PTEN* gene is also related with cell survival [39]. McLaughlin [37] described the employment of small strips for ovarian tissue preparation. As recently reported [27], ovarian fragmentation can induce follicular growth due to Hippo pathway disruption, but it would seem that the procedure does not work properly with currently used *in vitro* growth systems [40], although it has been successfully applied prior to *in vivo* development [27].

It is crucial to understand how PTEN inhibitors can affect the PTEN-Akt pathway in cancer patients since alterations in their expression have been described in hematologic malignancies [41–48]. These patients also represent the target population for the clinical application of IVA, followed by *in vitro* maturation, and also given the complexity of the oocyte maturation process.

Several studies that involve PTEN-Akt modulators have been developed with human tissue [27, 37, 49], including fresh and cryopreserved ovarian samples from cancer patients [26, 50]. To our knowledge however, this is the first report in which a comprehensive and systematic evaluation of follicular effects, hormone secretion, cell damage and the gene expression profile has been performed. We also aimed to develop our IVA protocol by using as few chemicals as possible, and also at low concentrations, to avoid adverse effects, but by using an effective dose that has been previously reported as effective [26].

When the fresh ovarian cortex was exposed to the PTEN inhibitor, it gave rise to a significant increase in the activated primordial follicles (FOXO3 nuclear export), the AMH expression in GC, and also in culture medium and E2 secretion (indicators of follicular development). As a result of the improvement in these processes, the percentage of growing follicles was also enhanced if compared to the control tissue. For previously cryopreserved-thawed ovarian tissue, a significant increase in activated primordial follicles, the AMH expression in GC, E2 secretion and the percentage of growing follicles was observed, as induced by the PTEN-inhibitor treatment when compared with the controls. This change in follicular subsets or populations did not affect follicular densities in either fresh or cryopreserved tissues. This finding reveals that the IVA protocol does not reduce the follicular pool, but promotes follicular development to growing stages. Besides, and as the Ki-67-positive staining revealed, primordial activated follicles are able to maintain their ability to proliferate.

The GC from both the fresh and cryopreserved activated tissues displayed a significantly increased AMH expression, an indicator of the secondary/preantral follicular phase, when compared to their own controls. Despite this enhanced AMH production having been previously described after PTEN-inhibition and Akt stimulation [26] in fresh ovarian tissue from mice, the effect has not yet been reported in fresh and cryopreserved human tissue when employing only a low dose of the PTEN inhibitor. It is noteworthy that a lower AMH expression level in GC was observed in previously cryopreserved tissue when compared to fresh samples. This AMH reduction phenomenon has already been described [51] as a result of cryopreservation procedures. However, our *in vitro* activation protocol was capable of restoring AMH production in frozen samples. In the present study, we cryopreserved samples by slow freezing following the methodology currently used in our clinical practice [29, 52] to evaluate if IVA can be a real option for our patients.

To elucidate whether the use of our short-term, low-concentration, ovary-specific treatment with a PTEN inhibitor induced any deleterious effect on follicles or the ovarian stroma, a complete study of cell damage after *in vitro* activation was conducted. After cell damage quantification and comparison with the cultured controls, we concluded that the use of a PTEN inhibition did not induce any structural alteration to the DNA of the stromal cells and follicles in fresh or cryopreserved samples.

Previous studies done with rodent ovaries have indicated that Tsc/mTORC1 and PTEN/PI3K signaling synergistically regulates the dormancy and activation of primordial follicles, and together, they ensure a proper female reproductive lifespan length [22]. Nonetheless when human ovarian cortex strips have been *in vitro* cultured with the mTOR inhibitor, a phagocytic program was activated, which resulted in the degradation of human oocytes [49]. Fortunately, the use of PTEN inhibitor bpv(piv) in our IVA protocol did not induce oocyte degradation, despite a minimum proportion of stromal cells being damaged due to the culture conditions (Fig 2). Moreover, the ovarian tissue cultured with a PTEN inhibitor stimulated the PTEN-PI3K-Akt pathway involved in oocyte maturation at the gene level without inducing cell damage in ovarian tissue, but tissue viability was maintained.

These findings demonstrate that IVA increases the pool of activated follicles. Thus it can be a first step to develop *in vitro* maturation protocols as an option to preserve fertility in cancer patients in whom ovarian transplant is avoided. Furthermore our results encourage us to continue developing IVA and *in vitro* growth procedures with dormant primordial follicles and to continue evaluating these results by several techniques.

## Supporting Information

S1 Fig Experimental design scheme. (TIF)

S2 Fig TUNEL staining. Four TUNEL and DAPI independent fields for each group have been included in order to shown the wide variability observed between samples of the same experimental group when apoptosis was analyzed.
(TIF)

S1 Table Exploratory study of densities and follicular populations between control samples. Tables shown the statistics obtained in the exploratory study performed for follicular densities and populations between the initial control and the cultured control samples.
(DOCX)

S2 Table Statistics from the gene expression analysis of *PTEN* pathway. (DOCX)
