# Supplementary figures and images for: Short-Term PTEN Inhibition Improves In Vitro Activation of Primordial Follicles, Preserves Follicular Viability, and Restores AMH Levels in Cryopreserved Ovarian Tissue From Cancer Patients
Source: PLoS One. 2015 May 29;10(5):e0127786. doi: 10.1371/journal.pone.0127786 (PMC4449215; doi:10.1371/journal.pone.0127786)

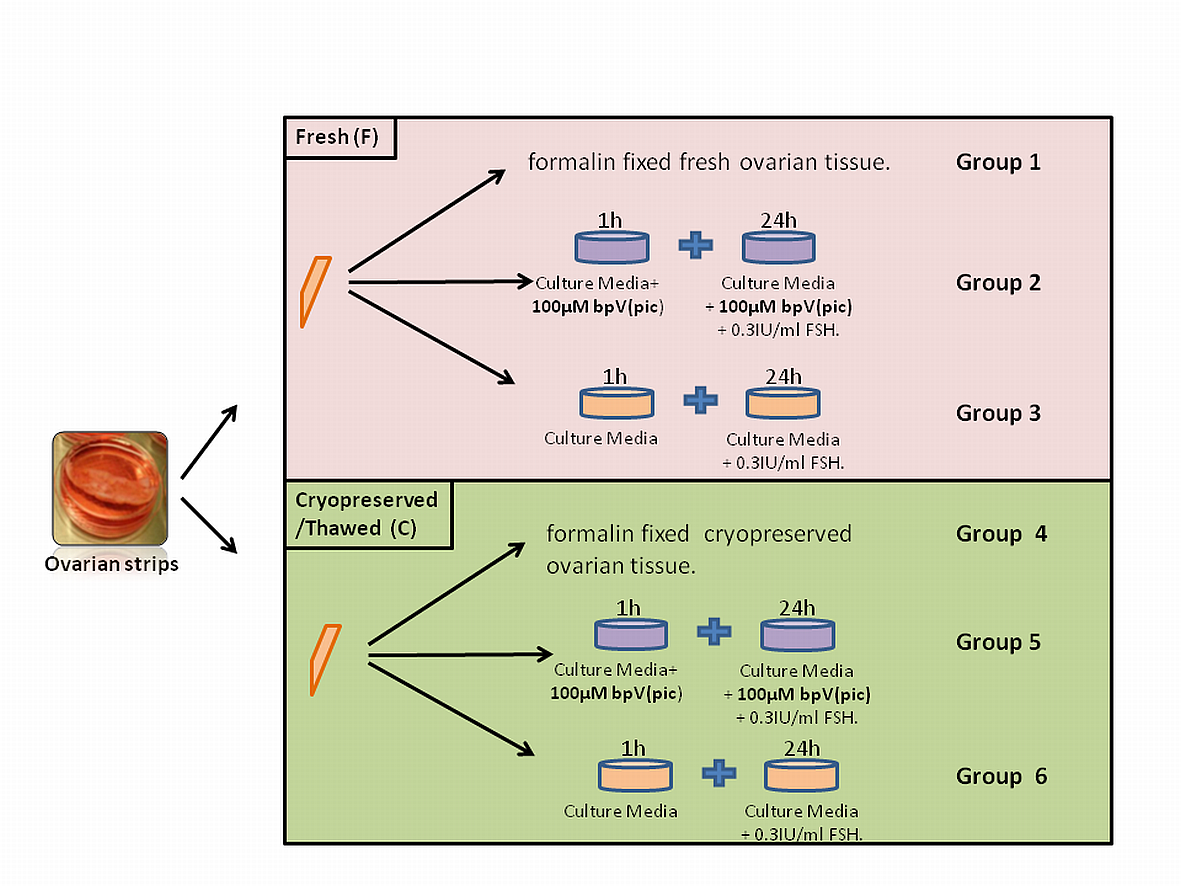

Supplement: S1 Fig — (TIF) [file pone.0127786.s001.tif]

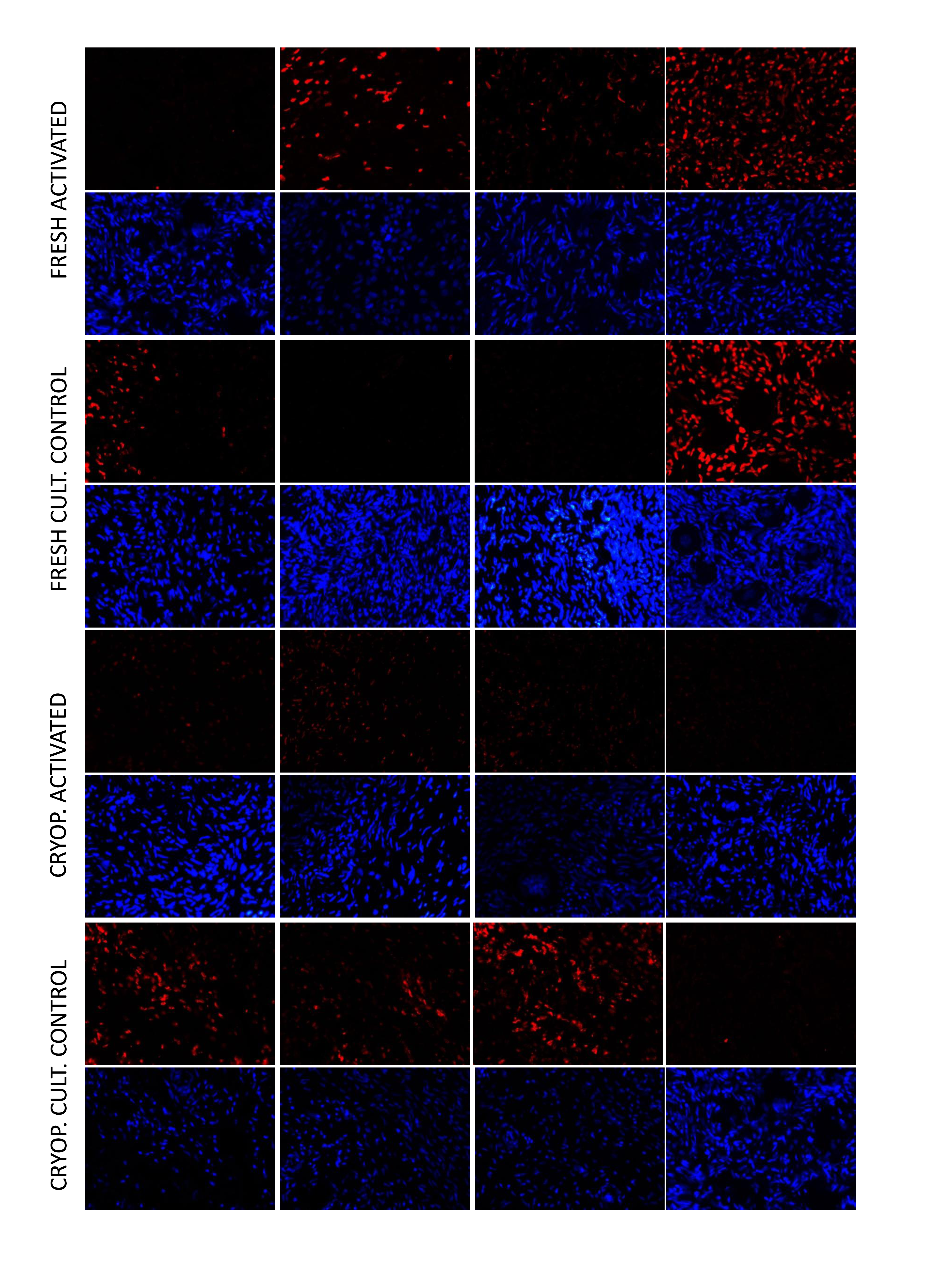

Supplement: S2 Fig — Four TUNEL and DAPI independent fields for each group have been included in order to shown the wide variability observed between samples of the same experimental group when apoptosis was analyzed. (TIF) [file pone.0127786.s002.tif]
