# Supplementary material for: Short-Term PTEN Inhibition Improves In Vitro Activation of Primordial Follicles, Preserves Follicular Viability, and Restores AMH Levels in Cryopreserved Ovarian Tissue From Cancer Patients
Source: PLoS One. 2015 May 29;10(5):e0127786. doi: 10.1371/journal.pone.0127786 (PMC4449215; doi:10.1371/journal.pone.0127786)
Supplement: S1 Table — Tables shown the statistics obtained in the exploratory study performed for follicular densities and populations between the initial control and the cultured control samples. (DOCX) [file pone.0127786.s003.docx]

| G1 vs. G3 | primordial fol/.mm2 | primary fol/.mm2 | secondary foll/mm2 | % Quiescent population |  |
| --- | --- | --- | --- | --- | --- |
| U de Mann-Whitney | 144,5 | 132,5 | 150 | 120 |  |
| W de Wilcoxon | 315,5 | 285,5 | 321 | 273 |  |
| Z | -0,282 | -0,685 | -0,151 | -0,58 |  |
| Sig. asintót. (bilateral) | 0,778 | 0,493 | 0,88 | 0,562 |  |
| Sig. exacta [2*(Sig. unilateral)] | **,782^a^** | **,503^a^** | **,935^a^** | **,581^a^** |  |
|  | | | | | |
| G4 vs. G6 | primordial fol/.mm2 | primary fol/.mm2 | secondary foll/mm2 | % Quiescent population |  |
| U de Mann-Whitney | 29 | 42 | 49,5 | 24 |  |
| W de Wilcoxon | 84 | 97 | 104,5 | 79 |  |
| Z | -1,594 | -0,614 | -0,073 | -1,989 |  |
| Sig. asintót. (bilateral) | 0,111 | 0,539 | 0,942 | 0,047 |  |
| Sig. exacta [2*(Sig. unilateral)] | **,123^a^** | **,579^a^** | **,971^a^** | **,052^a^** |  |

**Supplemental table 1.** Statistics from an exploratory study performed for follicular densities and populations between the initial control and the cultured control samples. As showed in the tables below, no differences were detected when compared with a paired t test.
