# Supplementary material for: Short-Term PTEN Inhibition Improves In Vitro Activation of Primordial Follicles, Preserves Follicular Viability, and Restores AMH Levels in Cryopreserved Ovarian Tissue From Cancer Patients
Source: PLoS One. 2015 May 29;10(5):e0127786. doi: 10.1371/journal.pone.0127786 (PMC4449215; doi:10.1371/journal.pone.0127786)
Supplement: S2 Table — (DOCX) [file pone.0127786.s004.docx]

| **Statistics gene expression analysis** | | | | | |
| --- | --- | --- | --- | --- | --- |
| Delta Ct |  | **Mean** | **SD** | **SEM** | **p value** |
| *Akt* | G1 | 6,202800 | 3,4793709 | 1,7396855 | 0,11 |
|  | G3 | 10,123167 | 1,6552379 | ,9556520 |  |
| *FOXO3* | G1 | 5,388400 | 2,8153895 | 1,4076947 | 0,07 |
|  | G3 | 9,346967 | 2,0950336 | 1,2095682 |  |
| *PI3K* | G1 | 8,753525 | 4,5564583 | 2,2782291 | 0,069 |
|  | G3 | 14,904267 | 1,2720361 | ,7344104 |  |
| *PTEN* | G1 | 6,944450 | 4,0552263 | 2,0276131 | 0,101 |
|  | G3 | 11,883933 | 1,1456671 | ,6614512 |  |
| *Akt* | G4 | 9,029460 | ,8236679 | ,3683555 | 0,67 |
|  | G6 | 9,727533 | 3,5328421 | 1,1776140 |  |
| *FOXO3* | G4 | 8,468140 | ,8479846 | ,3792303 | 0,43 |
|  | G6 | 9,391600 | 3,2442156 | 1,0814052 |  |
| *PI3K* | G4 | 13,394180 | 1,1309982 | ,5057978 | 0,332 |
|  | G6 | 11,537500 | 5,2286828 | 1,7428943 |  |
| *PTEN* | G4 | 12,169300 | 1,3842203 | ,6190421 | 0,333 |
|  | G6 | 10,510489 | 4,5366695 | 1,5122232 |  |

**Supplemental table 2. Statistics from the gene expression analysis of *PTEN* pathway.** Statistics from an exploratory study performed for gene expression between the initial control and the cultured control samples. As showed in the tables below, no differences were detected when compared with a paired t test.
